# Consistency of self‐reported and documented historical influenza vaccination status of US healthcare workers

**DOI:** 10.1111/irv.12988

**Published:** 2022-04-12

**Authors:** Annette K. Regan, Meredith G. Wesley, Manjusha Gaglani, Sara S. Kim, Laura J. Edwards, Kempapura Murthy, Zuha Jeddy, Allison L. Naleway, Brendan Flannery, Fatimah S. Dawood, Holly Groom

**Affiliations:** ^1^ School of Nursing and Health Professions University of San Francisco Orange California USA; ^2^ Department of Epidemiology, Fielding School of Public Health University of California Los Angeles Los Angeles California USA; ^3^ Wesfarmers Centre of Vaccines and Infectious Diseases Telethon Kids Institute Nedlands Western Australia Australia; ^4^ School of Public Health Texas A&M University College Station Texas USA; ^5^ Influenza Division Centers for Disease Control and Prevention Atlanta Georgia USA; ^6^ Abt Associates Atlanta Georgia USA; ^7^ Baylor Scott & White Health Temple Texas USA; ^8^ Texas A&M University College of Medicine College Station Texas USA; ^9^ The Center for Health Research Kaiser Permanente Northwest Portland Oregon USA

**Keywords:** healthcare personnel, influenza vaccines, self‐report, validity

## Abstract

**Background:**

Healthcare personnel (HCP) are a priority group for annual influenza vaccination. Few studies have assessed the validity of recall of prior influenza vaccination status among HCP, especially for more than one preceding season.

**Methods:**

Using data from a randomized controlled trial of influenza vaccination among 947 HCP from two US healthcare systems, we assessed agreement between participant self‐report and administrative record documentation of influenza vaccination status during the preceding five influenza seasons; kappa coefficients and sensitivity values were calculated. Administrative record documentation was considered the gold standard. Documented vaccination sources included electronic medical records, employee health records, outside immunization providers, and the state immunization information system.

**Results:**

Among 683 HCP with prior influenza immunization information, 89.7% (95% CI: 87.2%, 91.9%) of HCP were able to self‐report their vaccination status for the season preceding the survey. By the fifth preceding season, 82.6% (95% CI: 79.5%, 85.3%) of HCP were able to self‐report. Among HCP who self‐reported their vaccination status, agreement between self‐report and documented vaccination status ranged from 81.9% (95% CI: 77.2%, 86.7%) for the fifth season to 90.5% (95% CI: 87.2%, 93.9%) for the season preceding interview. HCP who received vaccine for only some of the preceding five seasons (18.3%) more commonly had ≥2 errors in their recall compared with those vaccinated all five preceding seasons (55.7% vs. 4.3%).

**Conclusions:**

Self‐reported vaccination status is a reliable source for historical influenza vaccination information among HCP who are consistently vaccinated but less reliable for those with a history of inconsistent vaccination.

## 1 INTRODUCTION

Healthcare personnel (HCP) experience a high degree of occupational exposure to influenza, and play a critical role in disrupting influenza transmission in healthcare settings.^1^, ^2^ For these reasons, HCP are considered a high priority group for influenza vaccination and an important population in which to assess annual influenza vaccine effectiveness and coverage.^3^ HCP are also a highly immunized population group. In the United States, as a result of employer‐based mandates and other vaccine promotion initiatives following the 2009 H1N1 pandemic, many HCP receive the influenza vaccine annually and have a history of repeated vaccination.^4^, ^5^ Because repeated vaccination may result in blunted immune responses to influenza vaccines over time,^6^, ^7^, ^8^ accurately accounting for prior influenza vaccine receipt over multiple seasons is important for studies of influenza vaccine effectiveness among HCP.

Influenza vaccination studies, including those among HCP, often rely on self‐reported vaccination status because of logistical challenges associated with verifying receipt of influenza vaccination. For example, most of the US annual influenza vaccination coverage estimates in HCP depend on self‐reported vaccination status as reported in the National Health Information Survey and the CDC Flu Panel Survey, published through FluVaxView annually.^9^, ^10^ Previous studies have shown that self‐report accurately captures the current season's influenza vaccination status.^11^, ^12^, ^13^, ^14^, ^15^ However, self‐report may be less accurate for determining prior season vaccination status.^11^ Misclassification of vaccination status may introduce bias in studies among frequently vaccinated groups, including HCP. Few studies have looked at the validity of recall of prior vaccination over multiple prior seasons or evaluated the validity of self‐report specifically in HCP, who may be differentially subject to social desirability or recall bias.^16^, ^17^

We evaluated the validity of historical self‐reported vaccination status over the preceding five seasons compared with administrative vaccination records among a cohort of HCP at two healthcare systems in the US.

## 2 MATERIAL AND METHODS

We conducted a cross‐sectional analysis of data collected from a multisite, randomized, open‐label influenza vaccine immunogenicity trial among HCP during the 2018–2019 and 2019–2020 Northern Hemisphere influenza seasons (Clinical Trials.gov Identifier NCT03722589). Study sites included two healthcare systems: Baylor Scott & White Health (BSWH) in Temple, Texas, and Kaiser Permanente Northwest (KPNW) in Portland, Oregon.^18^ Institutional Review Boards at both sites reviewed and approved the study protocol. Both participating sites had policies in place requiring or encouraging annual influenza vaccination of employees, with masking policies for unvaccinated HCP, dating back to the 2015–2016 influenza season (Table S1).^19^ HCP were screened for eligibility and enrolled if they were (i) aged 18–64 years, (ii) enrolled in their site's health network or reported that they received routine medical care with the site health system for at least 1 month, and (iii) consented. Participants included clinical professionals (i.e., physicians, dentists, nurse practitioners, physician assistants, nurses or midwives, allied health professionals, and pharmacists), clinical paraprofessionals (i.e., technicians, medical assistants, and patient transporter), and nonclinical support staff (i.e., front desk and administrative staff and research personnel).

Participants were enrolled during September–November of the 2018–2019 and 2019–2020 influenza seasons (Years 1 and 2, respectively). During enrollment, following informed consent, participants completed a brief survey which collected sociodemographic, health and occupational information, and self‐reported influenza vaccination during the current and five to six preceding seasons (Table S1). Some participants were in the study during both Years 1 and 2 and were asked about their influenza vaccination status and history at the start of each study season.

### 2.1 Influenza vaccination status

Participant‐reported vaccination status was verified against at least one of the following sources: (1) employee health immunization records; (2) medical information from immunization providers (i.e., pharmacies); (3) electronic medical record; (4) health insurance claims; and/or (5) state immunization information system (IIS) record (Table S1).

We categorized HCP as “consistent vaccinators” if they were vaccinated during all five seasons preceding the enrollment interview and “inconsistent vaccinators” if they were unvaccinated in at least one previous influenza season, based on documented vaccination status.

### 2.2 Statistical analysis

To ensure complete ascertainment of documented influenza vaccination status, this analysis was restricted to participants who completed the enrollment interview and were employed at the participating study site or enrolled in the health maintenance organization (HMO) or received medical care in the participating healthcare system for the 5 years preceding trial enrollment.

We calculated the percentage of HCP who provided complete self‐reported information on their influenza vaccination status for each of the five seasons preceding the interview (i.e., responded “yes” or “no” to receipt of vaccination). Among these participants, we calculated the absolute difference in vaccination rates for self‐report and verified records as well as the prevalence‐adjusted kappa coefficient and corresponding confidence intervals. We estimated sensitivity, specificity, and positive and negative predictive values for self‐report as compared with documented influenza vaccination status (gold standard) for each of the five seasons preceding the interview. For 147 participants recruited in Year 2, complete information was available for a sixth preceding influenza season; we performed additional comparisons for the sixth preceding season for this subgroup. For the subset of participating HCP who were interviewed in both Year 1 and Year 2, we estimated the percentage of participants who were able to self‐report their vaccination status and the degree of agreement between their self‐reported vaccination status in Year 1 and Year 2 using prevalence‐adjusted kappa coefficients.

We categorized participants as having “high degree of agreement” if they had no discrepancies, “moderate degree of agreement” if they had one discrepancy, and “low degree of agreement” if they had two or more discrepancies between their self‐reported and documented vaccination status for the five seasons preceding the interview. We compared the demographic, health and occupational information for participants by site, year of recruitment/interview, degree of agreement between self‐report and verified vaccination status, and completeness of vaccine information using the chi‐squared test for categorical variables and the Kruskal–Wallis test for continuous variables.

To evaluate how our inclusion criteria may have impacted study results, we performed several sensitivity analyses. First, we compared self‐reported and documented vaccination status for 801 HCP who were either employed or enrolled in the site HMO or received medical care in the participating healthcare system for the three seasons preceding the interview (instead of five seasons). Second, we compared self‐reported and documented vaccination status for 125 HCP classed as “inconsistent vaccinators,” who may experience more difficulty accurately recalling their historical vaccination status. Finally, we compared self‐reported and documented vaccination status for those 521 HCP who participated in Year 1 (2018–2019) only.

## 3 RESULTS

**FIGURE 1:**
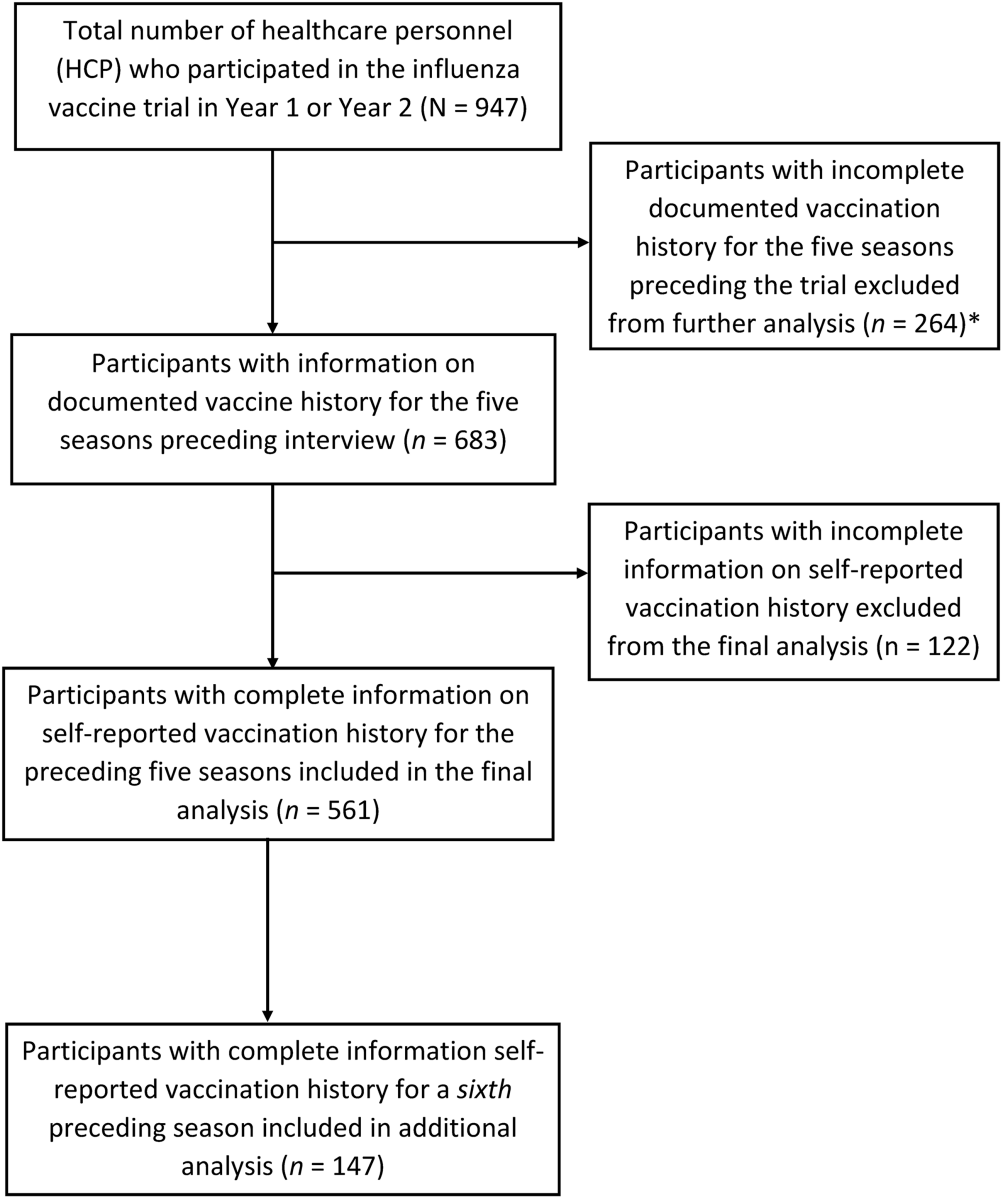
Selection of participants for comparison of self‐reported vaccination history to documented vaccination history among participating healthcare personnel—2018–2019 to 2019–2020. *Healthcare personnel were considered to have complete documented information if they were employed at the participating site or enrolled in the site health maintenance organization or received medical care in the participating healthcare system for the 5 years preceding trial enrollment

From the 947 HCP enrolled in the vaccine trial in Year 1 and Year 2, 683 had documented vaccination records available for the five seasons prior to enrollment and were included in the analysis (Figure 1). With exception to differences by site, age, and race/ethnicity, we identified no other differences between our study sample (*n* = 683) and other HCPs included in the trial (*n* = 264) (Table S2). Of the 683 included participants, 521 were enrolled in Year 1 and 162 HCP were enrolled in Year 2; 494 HCP contributed data in both years. Participants enrolled in Year 2 were more commonly recruited from the KPNW site and were less commonly categorized as clinical professionals (Table S3). Participants from the KPNW site were more commonly non‐Hispanic white and less commonly represented clinical professions (Table S4); 62% of participants were 45–64 years of age, 82% were female, 76% were non‐Hispanic white, and 53% had a college degree or higher education (Table 1); 62% were clinical professionals, 16% were clinical paraprofessionals, 16% were nonclinical support staff, and 6% were other personnel. Most (82%) participants were recorded as having received influenza vaccine in all five preceding seasons; clinical professionals were more commonly vaccinated in all five preceding seasons (Table S5).

**TABLE 1:** Characteristics of participating healthcare personnel (*N* = 683)—2018–2019 to 2019–2020

Characteristic	*N*	% (95% CI)
Age in years, mean (SD)	—	46.9 (9.7)
Age group		
18–44 years	262	38.4 (34.7, 42.0)
45–64 years	421	61.6 (58.0, 65.3)
Sex^a^		
Male	122	17.9 (15.0, 20.8)
Female	560	82.1 (79.2, 85.0)
Race/ethnicity^a^		
White, non‐Hispanic	518	76.3 (73.1, 79.5)
Black, non‐Hispanic	32	4.7 (3.1, 6.3)
Hispanic	79	11.6 (9.2, 14.1)
Other race, non‐Hispanic	50	7.4 (5.4, 9.3)
Educational attainment		
High school or less	45	6.6 (4.7, 8.5)
Some college/associate's degree	272	39.8 (36.1, 43.5)
Bachelor's degree	171	25.0 (21.8, 28.3)
Graduate degree	195	28.5 (25.1, 31.9)
Health characteristics		
BMI, mean (SD)	—	29.1 (7.0)
Cigarette smoker^a^	32	4.7 (3.1, 6.3)
Diagnosed or treated for chronic medical condition in past 12 months^a^	152	22.3 (19.2, 25.5)
Has immunosuppressive condition^b^	17	2.5 (1.3, 3.7)
Self‐rated general health status good or better^a^	669	98.2 (97.2, 99.2)
Employment characteristics		
Occupational category^a^		
Clinical professional	420	61.8 (58.1, 65.4)
Physician	72	10.6 (8.3, 12.9)
Dentist	2	0.3 (0.0, 0.7)
Nurse practitioner	11	1.6 (0.7, 2.6)
Physician assistant	4	0.6 (0.0, 1.2)
Nurse or midwife	238	35.0 (31.4, 38.6)
Allied health professional^c^	82	12.1 (9.6, 14.5)
Pharmacist	11	1.6 (0.7, 2.6)
Clinical paraprofessional	110	16.2 (13.4, 18.9)
Technicians	79	11.6 (9.2, 14.0)
Medical assistant	29	4.3 (2.7, 5.8)
Patient Transporter	2	0.3 (0.0, 0.7)
Nonclinical support staff	110	16.2 (13.4, 18.9)
Front desk and administrative staff	68	10.0 (7.7, 12.3)
Research personnel	42	6.2 (4.4, 8.0)
Other	40	5.9 (4.1, 7.70)
Frequency of vaccination^d^		
Consistent vaccinator	558	81.7 (81.3, 86.9)
Inconsistent vaccinator	125	18.3 (15.4, 21.2)
4/5 seasons	66	9.7 (7.4, 11.9)
<4/5 seasons	59	8.6 (6.5, 10.7)

^a^ One participant was missing information on sex, four were missing race/ethnicity, two were missing smoking status, two were missing physical activity, two were missing chronic disease diagnosis, two were missing self‐reported general health, and three were missing information on occupation.

^b^ Immunosuppressive conditions included a diagnosis of cancer with chemotherapy or radiation treatment, organ transplant, diagnosis of autoimmune disease, HIV or AIDS, or lymphopenia.

^c^ Includes therapists, nutritionists, phlebotomists, social workers, and psychologists.

^d^ A consistent vaccinator was defined as a participant who received an influenza vaccine all five seasons preceding the interview based on information in their HR, EMR, or vaccine immunization information system; an “inconsistent” vaccinator missed an influenza vaccination for at least one season during the five seasons preceding interview.

Most HCP were able to self‐report their vaccination status (i.e., provide a “yes” or “no” response) for the season preceding the interview (89.7%; 95% CI: 87.2%, 91.9%). However, the percent of participants who were able to self‐report their vaccination status declined to 82.6% (95% CI: 79.5%, 85.3%) by the fifth preceding season (Table 2). For the 147 participants with information on the sixth preceding season, 73.5% (95% CI: 65.5%, 80.4%) were able to self‐report their influenza vaccination status. Compared with participants who reported unknown vaccination status one or more preceding influenza seasons, participants who were able to consistently self‐report their vaccination status across all five preceding seasons were more commonly vaccinated during every preceding season, according to their vaccination record (83.9% vs. 71.3%, *P* < 0.001) (Table S6). When 494 participating HCP were asked to recall their vaccination status in the same season across two consecutive years (during their Year 1 interview and again during their Year 2 interview), we observed an 8.9% decline in the percentage of participants who were able to self‐report their vaccination status for the year prior to enrollment (i.e., the 2017–2018 season) (Table S7).

**TABLE 2:** Measures of agreement between self‐reported vaccination status and documented vaccination status for the five to six influenza seasons preceding interview among participating healthcare personnel (*N* = 683)—2018–2019 to 2019–2020

Influenza season	Able to self‐report influenza vaccine status		Self‐reported vaccination status	Documented vaccination status	Absolute difference^a^	Prevalence‐adjusted kappa coefficient^b^	Sensitivity	Specificity	Positive predictive value	Negative predictive value
	*N*	%	% (95% CI)	% (95% CI)	%	Coeff. (95% CI)	Value (95% CI)	Value (95% CI)	Value (95% CI)	Value (95% CI)
All sites (*N* = 683)										
Preceding season	613	89.7	97.1	97.9	−0.8	90.5 (87.2, 93.9)	97.2 (95.5, 98.3)	7.7 (0.2, 36.0)	98.0 (97.7, 98.3)	5.6 (0.8, 29.1)
2 seasons prior	611	89.5	99.5	92.8	6.7	86.6 (82.6, 90.5)	100 (99.3, 100)	6.8 (1.4, 18.7)	93.3 (92.7, 93.7)	—^c^
3 seasons prior	599	87.7	98.2	95.3	2.9	90.3 (86.9, 93.7)	98.9 (97.7, 99.6)	17.9 (6.1, 36.9)	96.1 (95.4, 96.7)	45.5 (21.3, 72.0)
4 seasons prior	584	85.5	98.1	92.5	5.6	86.0 (81.8, 90.1)	99.3 (98.1, 99.8)	15.9 (6.6, 30.1)	93.5 (92.7, 94.3)	63.6 (34.7, 85.2)
5 seasons prior	564	82.6	96.8	89.5	7.3	81.9 (77.2, 86.7)	99.0 (97.7, 99.7)	22.0 (12.3, 34.7)	91.6 (90.5, 92.6)	72.2 (49.0, 87.6)
6 seasons prior^d^	108	73.5	91.7	86.1	5.6	81.5 (70.5, 92.4)	97.9 (92.5, 99.7)	46.7 (21.3, 73.4)	91.9 (87.6, 94.8)	77.8 (44.5, 93.9)
Baylor Scott & White Health System (*N* = 318)										
Preceding season	276	86.8	96.7	96.4	0.3	93.1 (89.5, 95.8)	96.6 (93.7, 98.4)	—^c^	—^c^	—^c^
2 seasons prior	275	86.5	100	89.1	10.9	95.3 (92.0, 97.6)	100 (98.5, 100)	—^c^	—^c^	—^c^
3 seasons prior	271	85.2	99.3	93.0	6.3	84.5 (78.1, 90.9)	99.2 (97.2, 99.9)	—^c^	92.9 (89.3, 95.7)	—^c^
4 seasons prior	265	83.3	99.3	89.1	10.2	78.1 (70.6, 85.6)	99.6 (97.7, 100)	—^c^	89.3 (88.7, 90.0)	—^c^
5 seasons prior	259	81.5	98.8	88.0	10.8	73.7 (65.5, 82.0)	98.7 (96.2, 99.7)	—^c^	87.9 (87.7, 89.1)	—^c^
Kaiser Permanente Northwest (*N* = 365)										
Preceding season	337	92.3	97.3	99.1	−1.8	94.1 (90.4, 97.7)	97.6 (95.3, 99.0)	—	99.4 (98.7, 99.7)	11.1 (2.1, 41.6)
2 seasons prior	336	92.1	99.1	95.8	3.3	93.5 (89.7, 97.3)	100 (98.9, 100)	—	96.7 (95.7, 97.5)	—^c^
3 seasons prior	328	89.9	97.3	97.3	0	95.1 (91.8, 98.5)	98.7 (96.8, 99.7)	55.6 (21.2, 86.3)	98.7 (97.4, 99.4)	55.6 (28.7, 79.6)
4 seasons prior	319	87.4	97.2	95.3	1.9	92.5 (88.3, 96.7)	99.0 (97.1, 99.8)	40.0 (16.3, 67.7)	97.1 (95.7, 98.1)	66.7 (35.6, 87.9)
5 seasons prior	305	83.6	95.1	90.8	4.3	88.9 (83.7, 94.0)	99.3 (97.4, 99.9)	46.4 (27.5, 66.1)	94.8 (92.8, 96.3)	86.7 (60.7, 96.5)

^a^ Absolute difference in self‐reported vaccination status versus documented vaccination status.

^b^ Kappa coefficient showing percent agreement between self‐reported and documented influenza vaccination status.

^c^ Unable to estimate based on small numbers (*n* < 2).

^d^ Information on the sixth preceding season was available for 147 participants recruited in Year 2.

Agreement between self‐reported and documented vaccination status ranged from 81.9% (95% CI: 77.2%, 86.7%) in the fifth season preceding interview to 90.5% (95% CI: 87.2%, 93.9%) in season directly preceding interview (Table 2). For the 147 participants with information available on the sixth season preceding interview, we observed 81.5% (95% CI: 70.5%, 92.4%) agreement between self‐reported recall and documented vaccination status. Sensitivity values were high, ranging from 97.2% (95% CI: 95.5%, 98.3%) for the season directly preceding interview to 100% (95% CI: 99.3%, 100%) for two seasons prior to interview (Table 2). Positive predictive values exceeded 90%, ranging from 91.6% (95% CI: 90.5%, 92.6%) in the fifth season preceding interview to 98.0% (95% CI: 97.7%, 98.3%) in the season directly preceding interview. Estimates of specificity and negative predictive values had wide confidence intervals due to small numbers of unvaccinated participants. Recall and agreement patterns were similar for site stratified analyses (Table 2) and sensitivity analysis restricted to 521 Year 1 participants (Table S8) or the three seasons preceding interview in 801 HCP (Table S9).

**FIGURE 2:**
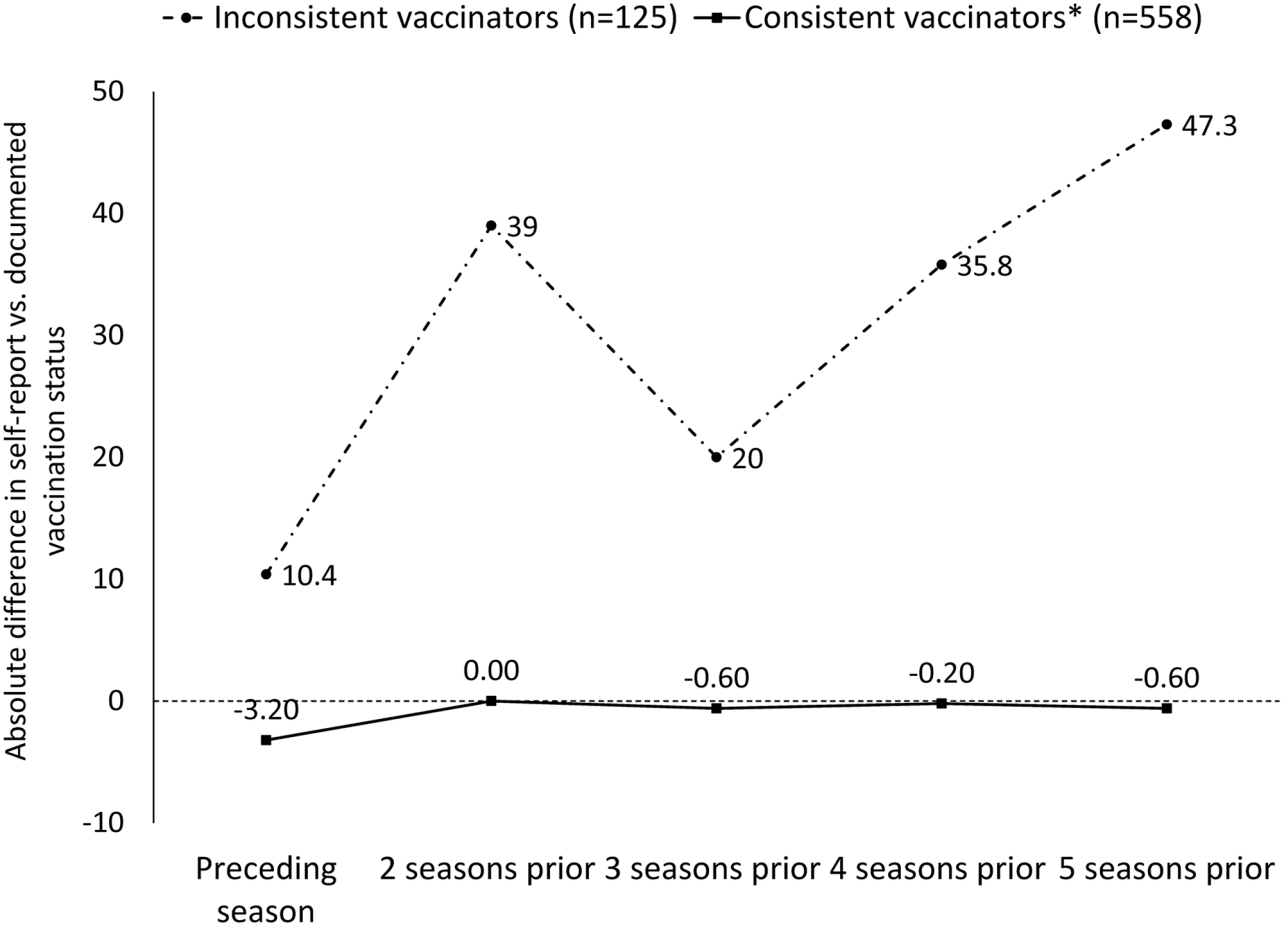
Absolute difference between self‐reported and documented influenza vaccination status for healthcare personnel, by frequency of vaccination—2018–2019 to 2019–2020. *A “consistent” vaccinator was defined as a participant who received an influenza vaccine during all five influenza seasons preceding interview based on information in their HR, EMR, or vaccine register; an “inconsistent” vaccinator was unvaccinated for at least one season during the five seasons preceding interview

More than 70% of both “inconsistent” and “consistent” vaccinators were able to report their vaccination status for each of the five seasons preceding interview (Figure S1). However, the absolute difference between “inconsistent” vaccinators' self‐reported and documented influenza vaccination status increased from 10% for the season preceding interview to 47% for the fifth season preceding interview (Figure 2). The absolute difference between “consistent” vaccinators' self‐reported and documented influenza vaccination status did not exceed 5% for all five preceding seasons (Figure 2). Adjusted kappa coefficients were high for “inconsistent vaccinators” for the year preceding interview (75.5%; 95% CI: 63.0%, 88.0%), and agreement was low (<50%) for two or more seasons preceding interview (Table S10).

Participants who had a high degree of agreement between their recall and documented vaccination status for the five preceding seasons were all classified as consistently vaccinated, were predominantly clinical professionals (69%) and reported good or better general health (99%) (Table 3). Participants who had the most discrepancies in accurately recalling their vaccination status were commonly classed as inconsistent vaccinators (73%) and were less frequently clinical professionals (54%) (*P* = 0.03) (Table 3).

**TABLE 3:** Characteristics of those with high, moderate and low degree of agreement between self‐report and documented influenza vaccination among participating healthcare personnel (*N* = 613)—2018–2019 to 2019–2020

Characteristic	High degree of agreement^a^ (*N* = 453)			Moderate degree of agreement^a^ (*N* = 79)			Low degree of agreement^a^ (*N* = 81)			*P* value^b^
	*N*	%	(95% CI)	*N*	%	(95% CI)	*N*	%	(95% CI)	
Study site										0.18
Baylor Scott & White Health System	199	43.9	(39.3, 48.5)	33	41.8	(30.9, 52.7)	44	54.3	(43.4, 65.2)	
Kaiser Permanente Northwest	254	56.1	(51.5, 60.7)	46	58.2	(47.3., 69.1)	37	45.7	(34.8, 56.5)	
Age in years, mean (SD)	—	47.2	(9.5)	—	48.3	(9.1)	—	45.2	(10.0)	0.15
Age group										0.41
18–44 years	168	37.1	(32.6, 41.5)	28	35.4	(24.9, 46.0)	36	44.4	(33.6, 55.3)	
45–64 years	285	62.9	(58.5, 67.4)	51	64.5	(54.0, 75.1)	45	55.5	(44.7, 66.4)	
Sex^c^										0.14
Male	78	17.2	(13.7, 20.7)	14	17.9	(9.4, 26.5)	15	18.5	(10.0, 27.0)	
Female	375	82.8	(72.3, 86.3)	64	82.1	(73.5, 90.6)	66	81.5	(73.0, 90.0)	
Race/ethnicity^c^										0.11
White, non‐Hispanic	361	80.0	(76.3, 83.7)	57	74.0	(64.2, 83.8)	59	72.8	(63.1, 82.5)	
Black, non‐Hispanic	13	2.9	(1.3, 4.4)	3	3.9	(0.0, 8.2)	8	9.9	(3.4, 16.4)	
Hispanic	45	10.0	(7.2, 12.7)	10	13.0	(5.5, 20.5)	9	11.1	(4.2, 18.0)	
Other race, non‐Hispanic	32	7.1	(4.7, 9.5)	7	9.1	(2.7, 15.5)	5	6.2	(0.9, 11.4)	
Educational attainment										0.52
High school or less	20	4.4	(2.5, 6.3)	2	2.5	(0.0, 6.0)	4	4.9	(0.2, 9.7)	
Some college/associate's degree	168	37.1	(32.6, 41.5)	36	45.6	(34.5, 56.6)	36	44.4	(33.6, 55.3)	
Bachelor's degree	120	26.5	(22.4, 30.6)	19	24.1	(14.6, 33.5)	23	28.4	(18.5, 38.2)	
Graduate degree	145	32.0	(27.7, 36.3)	22	27.8	(17.9, 37.8)	18	22.2	(13.1, 31.3)	
Average number of household contacts, mean (SD)	—	2.1	(1.3)	—	2.0	(1.3)	—	2.0	(1.3)	0.40
Health characteristics										
BMI, mean (SD)	—	28.6	(6.7)	—	27.9	(5.7)	—	31.2	(8.2)	0.03
Cigarette smoker	17	3.7	(2.0, 5.5)	5	6.3	(0.9, 11.7)	2	2.5	(0.0, 5.9)	0.43
Diagnosed or treated for chronic medical condition in past 12 months	111	24.5	(20.5, 28.5)	19	24.1	(14.6, 33.5)	12	14.8	(7.1, 22.6)	0.16
Has immunosuppressive condition^d^	12	2.6	(1.2, 4.1)	3	3.8	(0.0, 8.0)	0			0.26
Self‐rated general health status good or better	450	99.3	(98.6, 100)	76	96.2	(92.0, 100)	78	96.3	(92.2, 100)	0.02
Occupational category^c^										0.03
Clinical professional	310	68.6	(64.3, 72.9)	42	53.2	(42.1, 64.2)	43	53.7	(42.8, 64.7)	
Physician	61	13.5	(10.3, 16.7)	7	8.9	(2.6, 15.1)	1	1.3	(0.0, 3.7)	
Dentist	0	0.0	—	2	2.5	(0.0, 6.0)	0	0.0	—	
Nurse practitioner	8	1.8	(0.5, 3.0)	3	3.8	(0.0, 8.0)	0	0.0	—	
Physician assistant	4	0.9	(0.0, 1.7)	0	0.0	—	0	0.0	—	
Nurse or midwife	177	39.1	(34.6, 43.7)	18	22.8	(13.5, 32.1)	30	37.5	(26.9, 48.1)	
Allied health professional^e^	52	11.5	(8.5, 14.5)	11	13.9	(6.3, 21.6)	11	13.7	(6.2, 21.3)	
Pharmacist	8	1.8	(0.5, 3.0)	1	1.3	(0.0, 3.7)	1	1.3	(0.0, 3.7)	
Clinical paraprofessional	59	13.1	(9.9, 16.2)	16	20.3	(11.4, 29.1)	13	16.3	(8.1, 24.3)	
Technicians	44	9.7	(7.0, 12.5)	12	15.2	(7.3, 23.1)	9	11.3	(4.3, 18.2)	
Medical assistant	15	3.3	(1.7, 5.0)	4	5.1	(0.2, 9.9)	4	5.0	(0.2, 9.8)	
Nonclinical support staff	62	13.7	(10.5, 16.9)	18	22.8	(13.5, 32.1)	19	23.7	(14.4, 33.1)	
Front desk and administrative staff	38	8.4	(5.8, 11.0)	13	16.5	(8.2, 24.7)	10	12.5	(5.2, 19.8)	
Research personnel	24	5.3	(3.2, 7.4)	5	6.3	(0.9, 11.7)	9	11.3	(4.3, 18.1)	
Other personnel	21	4.6	(2.7, 6.6)	3	3.8	(0.0, 8.0)	5	6.3	(0.9, 11.6)	
Frequency of vaccination^f^										<0.001
Consistent vaccinator	453	100.0	—	32	40.5	(29.7, 51.4)	22	27.2	(17.4, 36.9)	
Inconsistent vaccinator	0	0.0	—	47	59.5	(48.6, 70.3)	59	72.8	(63.1, 82.5)	
4/5 seasons	0	0.0	—	47	59.5	(48.6, 70.3)	9	11.1	(4.2, 18.0)	
<4/5 seasons	0	0.0	—	0	0.0	—	50	61.7	(51.1, 72.3)	

Abbreviation: CI, confidence interval.

^a^ High degree of agreement were individuals with zero discrepancy, moderate had one discrepancy, and low agreement had two or more discrepancies over the preceding five seasons.

^b^ *P* value derived from chi‐squared test for categorical variables and Kruskal–Wallis test for continuous variables.

^c^ One individual missing sex, four missing race/ethnicity, and two missing occupation.

^d^ Immunosuppressive conditions included a diagnosis of cancer with chemotherapy or radiation treatment, organ transplant, diagnosis of autoimmune disease, HIV or AIDS, or lymphopenia.

^e^ Includes therapists, nutritionists, phlebotomists, social workers, and psychologists.

^f^ A consistent vaccinator was defined as a participant who received an influenza vaccine all five seasons based on information in their HR, EMR, or vaccine register; an “inconsistent” vaccinator missed an influenza vaccination for at least one season during the five seasons preceding interview.

## 4 DISCUSSION

In our study, nearly 82% of participating HCP had validated influenza vaccination in each of the five preceding seasons, leaving 18% with inconsistent vaccination histories. Those with inconsistent vaccination were split between those who received influenza vaccinations in four of the five preceding seasons (9.6%) and those who were vaccinated in three or fewer of the five prior influenza seasons (8.6%). Occupational category did factor into observed differences in vaccination histories, as individuals who worked as clinical professionals were more likely to be categorized as consistently vaccinated, compared with other HCP types (85% vs. 76%). This finding highlights the possibility that HCP role may be correlated with annual vaccination patterns.

Not all participants were able or willing to self‐report their vaccination histories. While 82% did provide these details, we found that lower proportions of those with inconsistent vaccination (per validated record review) and those in HCP roles that were not defined as “clinical professional” self‐reported their influenza vaccination histories. Inconsistency of uptake from year to year may present challenges when attempting to recall past years, particularly those in the more distant past. The interplay between these factors may be important to consider when evaluating the validity of recall in place of documented vaccination histories for the influenza vaccine.

Overall, recall accuracy was high, ranging from 87% to 91% agreement when compared with verified administrative records for the prior three influenza seasons. Accuracy began to diminish in the fourth season but remained above 80%, overall, even in the fifth preceding season. It is important to note, however, that this high level of accuracy was driven largely by individuals with validated consistent annual vaccination, representing 82% of our participants, as only they accurately self‐reported vaccination history in all five prior influenza seasons. Consistent vaccinators were also more likely to provide self‐reported vaccination histories (84%, compared with 72% of those with inconsistent vaccination histories), and given our study population, strongly influenced our assessments of recall accuracy. When we looked at accuracy of recall among just those 125 participants with inconsistent vaccination histories, the accuracy of recall decreased markedly, from 88% in the immediately preceding season, to 58% in two seasons prior. It is possible that the impact of more explicit policies for HCP influenza vaccination that are being adopted in healthcare facilities in the United States^20^ are contributing to more consistent annual vaccination uptake. Even in states without laws mandating influenza vaccination of HCP (such as Texas and Oregon),^21^ facility efforts to promote vaccination paired with policies such as masking could increase the consistency of vaccination, making it easier to recall vaccination history in this setting.^5^, ^22^, ^23^, ^24^ However, variations in consistent vaccination patterns among HCP by role or employment duration may be important factors when considering the impact of using self‐reported vaccination status.

Even with high and sustained accuracy, we observed a tendency to overestimate vaccine uptake in past years. This effect was true across all study participants, beginning at two seasons in the past, but never exceeded 7% across all five seasons. Among the inconsistently vaccinated group, self‐reported vaccination was 20% to 47% higher, compared with verified vaccination, for influenza seasons 2 to 5 years in the past. Here, we see that self‐report is subject to overestimation once individuals are recalling for influenza seasons two or more years in the past, and the magnitude of that overestimation is even greater when respondents have been inconsistently vaccinated over prior influenza season. The idea that people are more likely to overestimate vaccination has been commonly reported in studies of influenza vaccine recall.^11^, ^12^, ^15^, ^25^ Overestimating prior vaccination may be more common among HCP who face pressure to vaccinate annually and may experience heightened susceptibility to social desirability bias^16^, ^17^; however, the fact that overestimation seems to be associated with inconsistent vaccination patterns and/or increasing number of years since vaccination suggests that this overestimation may simply be an issue of incorrect recall.

Few studies have evaluated the accuracy of self‐reported vaccination status among HCP, even though they represent a group where historical information on vaccination can be highly useful. Studies limited to just one prior influenza season report high rates of agreement between self‐report and validated vaccination status.^25^, ^26^ One such study, focusing on community‐based participants recruited as part of an influenza vaccine effectiveness study, found that accurate recall dropped from 98% in the current season to 93% percent agreement in the season prior.^11^ A second study, also drawing from a study of influenza vaccine effectiveness in 2008, found an overall 93% agreement between self‐reported vaccination status compared with documented vaccination status in an IIS over two consecutive influenza seasons.^12^ A third study, spanning the 2007–2009 influenza seasons in Spain, validated self‐reported vaccination among HCP in the current and three previous seasons. The investigators found that the validity of self‐report was stable, at 72% agreement, for up to two prior seasons, but diminished substantially by the third prior season (36% agreement), suggesting two prior years as a potential cut point for vaccine evaluation studies.^15^ There were, however, very low rates of validated influenza vaccination in the Spanish HCP cohort, ranging from 14% to 29% between 2007 and 2009 which means most HCP were not routinely vaccinated and may have had a harder time with recall. Our sub‐analysis of inconsistently vaccinated study participants revealed very similar findings and validates the limitations that come with relying on self‐report of vaccination beyond just one prior influenza season among HCP who are not required to or do not receive annual influenza vaccines.

Strengths of this study include our ability to analyze data for a large sample of HCP using multiple sources of information, including self‐report, electronic medical records, human resources records, and state IIS. Second, because we conducted this study with HCP, we were able to include a well‐defined lookback period based on employment and other records. This allowed the opportunity to verify information over a 5‐ to 6‐year period. Finally, we were able to measure agreement between self‐reported and documented vaccination status across two distinct health systems. Despite different health systems and some differences in ability to recall vaccination status, results across the two sites showed mostly similar patterns; these results suggest replicability of our findings. Limitations include differential ascertainment. Our “validation” source for influenza vaccination may have been incomplete or differentially accurate by site. However, because the primary analysis was restricted to participants who had either been employed at the participating site or enrolled in the participating healthcare system or HMO for the 5 years preceding the study interview, we would expect to have comprehensive data overall and would not expect differences in available vaccination history for any particular group or subset of the study population. Our study population was mostly female (81%) and overrepresented older HCP. Furthermore, because HCP are a highly immunized group, the majority of our sample were vaccinated every season (i.e., “consistent” vaccinators). Results from this study may not be generalizable to other study populations. Finally, this was a highly vaccinated cohort of HCP. As a result, numbers of unvaccinated HCPs were too small to estimate NPV and specificity.

### 4.1 Conclusions

Based on analysis of self‐reported influenza vaccination status from HCP across five preceding influenza seasons, we found that recall was highly accurate when compared with verified information sources. However, these results were mostly observed for those immunized every season, and beyond one prior season, all individuals tended to overestimate influenza vaccine uptake. The magnitude of that overestimation noticeably increased among those with inconsistent influenza vaccine uptake across the five prior years. In the absence of available documentation such as electronic medical records or IIS data, self‐report is an acceptable method of data collection for studies aiming to measure historical exposure to influenza vaccines. To minimize potential misclassification of vaccination status, especially in inconsistently vaccinated individuals, reliance on self‐report should be restricted to one prior influenza season unless an annual vaccination mandate is in place.

## AUTHOR CONTRIBUTIONS

**Annette K. Regan:** Conceptualization; formal analysis; methodology. **Meredith G. Wesley:** Methodology. **Manjusha Gaglani:** Data curation; funding acquisition; methodology; validation. **Sara S. Kim:** Project administration. **Laura J. Edwards:** Data curation. **Kempapura Murthy:** Data curation; validation. **Zuha Jeddy:** Data curation. **Allison L. Naleway:** Data curation; funding acquisition. **Brendan Flannery:** Project administration. **Fatimah S. Dawood:** Project administration. **Holly Groom:** Data curation; methodology.

## DISCLAIMER

The findings and conclusions in this report are those of the authors and do not necessarily represent the views of the Centers for Disease Control and Prevention.

### PEER REVIEW

The peer review history for this article is available at https://publons.com/publon/10.1111/irv.12988.

## Supporting information


**Table S1.** Summary of documented and self‐reported sources of influenza vaccine information for participating healthcare personnel, by study site – 2018‐19 to 2019‐20.
**Table S2**. Characteristics of participating healthcare personnel employed at or enrolled in the participating healthcare system for the five years preceding interview (Included Participants) vs. those employed or enrolled in the healthcare system <5 years preceding interview (Excluded Participants) – 2018‐19 to 2019‐20.
**Table S3**. Characteristics of participating healthcare personnel, by year of enrolment – 2018‐19 to 2019‐20.
**Table S4.** Characteristics of participating healthcare personnel, by study site – 2018‐19 to 2019‐20.
**Table S5.** Characteristics of participating healthcare personnel, by occupational category – 2018‐19 to 2019‐20.
**Table S6**. Characteristics of healthcare personnel (N=683) with known and uncertain self‐reported influenza vaccination status for the five influenza seasons preceding interview – 2018‐19 to 2019‐20.
**Table S7**. Measures of agreement between self‐reported vaccination status for healthcare personnel (N=494)* as measured across two consecutive years of interview – 2018‐19 to 2019‐20.
**Table S8**. Sensitivity analysis of agreement between self‐reported and documented influenza vaccination status for healthcare personnel participating in Year 1 only (N=521) – 2018‐19.
**Table S9**. Sensitivity analysis of agreement between self‐reported and documented influenza vaccination status during the three years prior to interview among participating healthcare personnel (N=801) – 2018‐19 to 2019‐20.
**Table S10.** Sensitivity analysis of agreement between self‐reported and documented influenza vaccination status for healthcare personnel who did not receive an influenza vaccine in all five seasons preceding enrolment (i.e., “inconsistent vaccinators*) (N=125) – 2018‐19 to 2019‐20.
**Figure S1.** Percent of healthcare personnel self‐reporting their influenza vaccination status for the five preceding influenza seasons, by frequency of vaccination – 2018‐19 to 2019‐20.

## Data Availability

Data were collected as part of a randomized controlled trial (NCT03722589). Individual participant data can be made available to other researchers upon request.
