# Supplementary material for: Consistency of self‐reported and documented historical influenza vaccination status of US healthcare workers
Source: Influenza Other Respir Viruses. 2022 Apr 12;16(5):881–90. doi: 10.1111/irv.12988 (PMC9343323; doi:10.1111/irv.12988)
Supplement: Supplementary file 1 — Table S1. Summary of documented and self‐reported sources of influenza vaccine information for participating healthcare personnel, by study site – 2018‐19 to 2019‐20. Table S2. Characteristics of participating healthcare personnel employed at or enrolled in the participating healthcare system for the five years preceding interview (Included Participants) vs. those employed or enrolled in the healthcare system <5 years preceding interview (Excluded Participants) – 2018‐19 to 2019‐20. Table S3. Characteristics of participating healthcare personnel, by year of enrolment – 2018‐19 to 2019‐20. Table S4. Characteristics of participating healthcare personnel, by study site – 2018‐19 to 2019‐20. Table S5. Characteristics of participating healthcare personnel, by occupational category – 2018‐19 to 2019‐20. Table S6. Characteristics of healthcare personnel (N=683) with known and uncertain self‐reported influenza vaccination status for the five influenza seasons preceding interview – 2018‐19 to 2019‐20. Table S7. Measures of agreement between self‐reported vaccination status for healthcare personnel (N=494)* as measured across two consecutive years of interview – 2018‐19 to 2019‐20. Table S8. Sensitivity analysis of agreement between self‐reported and documented influenza vaccination status for healthcare personnel participating in Year 1 only (N=521) – 2018‐19. Table S9. Sensitivity analysis of agreement between self‐reported and documented influenza vaccination status during the three years prior to interview among participating healthcare personnel (N=801) – 2018‐19 to 2019‐20. Table S10. Sensitivity analysis of agreement between self‐reported and documented influenza vaccination status for healthcare personnel who did not receive an influenza vaccine in all five seasons preceding enrolment (i.e., “inconsistent vaccinators*) (N=125) – 2018‐19 to 2019‐20. Figure S1. Percent of healthcare personnel self‐reporting their influenza vaccination status for the five preceding influ [file IRV-16-881-s001.docx]

**Supplement to**: Regan, A.K., Wesley, M. G., Gaglani, M., Kim, S., Edwards, L., Kempapura, M., Jeddy, Z., Naleway, A., Flannery, B., Dawood, F.S., Groom, H. Consistency of self-reported and documented historical influenza vaccination status of US healthcare workers.

**Table S1.** Summary of documented and self-reported sources of influenza vaccine information for participating healthcare personnel, by study site – 2018-19 to 2019-20.

|  | **Baylor Scott and White Health System** | **Kaiser Permanente Northwest** |
| --- | --- | --- |
| **Record Verification Sources** | | |
| Employee Health immunization records | X |  |
| Medical release of information from outside immunization providers | X |  |
| Health system electronic medical records * | X | X |
| Health insurance claims* | X | X |
| State immunization information system† | X | X |
| **Survey item collecting self-reported vaccination status** | | |
| The following questions will ask about whether you received the seasonal influenza vaccine during each of the past 5 influenza seasons. Did you receive the influenza vaccine during the:  2017 – 2018 influenza season?  Yes   No   Don’t know/can’t remember  2016 – 2015 influenza season?  Yes   No   Don’t know/can’t remember  2015 – 2014 influenza season?  Yes   No   Don’t know/can’t remember  2014 – 2015 influenza season?  Yes   No   Don’t know/can’t remember  2013 – 2014 influenza season?  Yes   No   Don’t know/can’t remember | X | X |

*For those enrolled in employer-provided medical plan or receiving medical care in the local health system

†The state immunization information system is opt-in in TX (Baylor Scott and White Health System) and opt-out for OR (Kaiser Permanente Northwest).

**Table S2**. Characteristics of participating healthcare personnel employed at or enrolled in the participating healthcare system for the five years preceding interview (Included Participants) vs. those employed or enrolled in the healthcare system <5 years preceding interview (Excluded Participants) – 2018-19 to 2019-20.

| **Characteristic** | **Included Participants (N=683)** | | | **Excluded Participants (N=264)** | | | **P-value*** |
| --- | --- | --- | --- | --- | --- | --- | --- |
|  | **N** | **%** | **(95% CI)** | **N** | **%** | **(95% CI)** |  |
| **Study site** |  |  |  |  |  |  | <0.001 |
| Baylor Scott & White | 318 | 46.5 | (42.8, 50.3) | 170 | 64.4 | (58.6, 70.2) |  |
| Kaiser Permanente Northwest | 365 | 53.4 | (49.7, 57.2) | 94 | 35.7 | (29.8, 41.4) |  |
| **Age in years, mean (SD)** | --- | 46.9 | (9.7) | --- | 36.7 | (10.6) | <0.001 |
| **Age group** |  |  |  |  |  |  | <0.001 |
| 18-44 years | 262 | 38.4 | (34.7, 42.0) | 198 | 75.0 | (69.8, 80.2) |  |
| 45-64 years | 421 | 61.6 | (58.0, 65.3) | 66 | 25.0 | (19.8, 30.2) |  |
| **Sex^†^** |  |  |  |  |  |  | 0.47 |
| Male | 122 | 17.9 | (15.0, 20.8) | 52 | 19.9 | (15.1, 24.8) |  |
| Female | 560 | 82.1 | (79.2, 85.0) | 209 | 80.1 | (75.2, 84.9) |  |
| **Race/ethnicity^†^** |  |  |  |  |  |  | 0.02 |
| White, non-Hispanic | 518 | 76.3 | (73.1, 79.5) | 171 | 66.5 | (60.7, 72.3) |  |
| Black, non-Hispanic | 32 | 4.7 | (3.1, 6.3) | 21 | 8.2 | (4.8, 11.5) |  |
| Hispanic | 79 | 11.6 | (9.2, 14.1) | 43 | 16.7 | (12.1, 21.3) |  |
| Other race, non-Hispanic | 50 | 7.4 | (5.4, 9.3) | 22 | 8.6 | (5.1, 12.0) |  |
| **Educational attainment** |  |  |  |  |  |  | 0.38 |
| High school or less | 45 | 6.6 | (4.7, 8.5) | 18 | 6.8 | (3.8, 9.9) |  |
| Some college / Associate's degree | 272 | 39.8 | (36.1, 43.5) | 93 | 35.2 | (29.5, 41.0) |  |
| Bachelor's degree | 171 | 25.0 | (21.8, 28.3) | 80 | 30.3 | (24.7, 35.9) |  |
| Graduate degree | 195 | 28.5 | (25.1, 31.9) | 73 | 27.7 | (22.2, 33.1) |  |
| **Health characteristics** |  |  |  |  |  |  |  |
| BMI, mean (SD) | --- | 29.1 | (7.0) | --- | 29.0 | (7.3) | 0.67 |
| Cigarette smoker**^†^** | 32 | 4.7 | (3.1, 6.3) | 5 | 1.9 | (0.3, 3.6) | 0.05 |
| Diagnosed or treated for chronic medical condition**^†^** | 152 | 22.3 | (19.2, 25.5) | 46 | 17.8 | (13.1, 22.4) | 0.13 |
| Has immunosuppressive condition^§^ | 17 | 2.5 | (1.3, 3.7) | 4 | 1.5 | (0.0, 3.0) | 0.36 |
| Self-rated general health status good or better**^†^** | 669 | 98.2 | (97.2, 99.2) | 256 | 98.8 | (97.5, 100) | 0.51 |
| **Employment characteristics** |  |  |  |  |  |  |  |
| Occupational category**^†^** |  |  |  |  |  |  | 0.08 |
| *Clinical professional* | 420 | 61.8 | (58.1, 65.4) | 166 | 63.8 | (58.0, 69.7) |  |
| Physician | 72 | 10.6 | (8.3, 12.9) | 23 | 8.8 | (5.4, 12.3) |  |
| Dentist | 2 | 0.3 | (0.0, 0.7) | 0 | 0.0 | --- |  |
| Nurse practitioner | 11 | 1.6 | (0.7, 2.6) | 7 | 2.7 | (0.7, 4.7) |  |
| Physician assistant | 4 | 0.6 | (0.0, 1.2) | 4 | 1.5 | (0.0, 3.0) |  |
| Nurse or midwife | 238 | 35.0 | (31.4, 38.6) | 102 | 39.2 | (33.3, 45.2) |  |
| Allied health professional^⁋^ | 82 | 12.1 | (9.6, 14.5) | 28 | 10.8 | (7.0, 14.5) |  |
| Pharmacist | 11 | 1.6 | (0.7, 2.6) | 2 | 0.8 | (0.0, 1.8) |  |
| *Clinical paraprofessional* | 110 | 16.2 | (13.4, 18.9) | 35 | 13.5 | (9.3, 17.6) |  |
| Technicians | 79 | 11.6 | (9.2, 14.0) | 21 | 8.1 | (4.8, 11.4) |  |
| Medical assistant | 29 | 4.3 | (2.7, 5.8) | 14 | 5.4 | (2.6, 8.1) |  |
| Patient Transporter | 2 | 0.3 | (0.0, 0.7) | 0 | 0.0 | --- |  |
| *Non-clinical support staff* | 110 | 16.2 | (13.4, 18.9) | 31 | 11.9 | (8.0, 15.9) |  |
| Front Desk & Administrative Staff | 68 | 10.0 | (7.7, 12.3) | 16 | 6.1 | (3.2, 9.1) |  |
| Research personnel | 42 | 6.2 | (4.4, 8.0) | 15 | 5.8 | (2.9, 8.6) |  |
| *Other* | 40 | 5.9 | (4.1, 7.7) | 28 | 10.8 | (7.0, 14.5) |  |

* P-value derived from Chi-squared test for categorical variables and Kruskal Wallis test for continuous variables.

^†^ Information was missing for sex (n=4), race/ethnicity (n=11), smoking status (n=7), physical activity (n=7), diagnosis with a chronic disease (n=7), self-reported general health (n=7), and occupational category (n=7).

^§^ immunosuppressive conditions included a diagnosis of cancer with chemotherapy or radiation treatment, organ transplant, diagnosis of autoimmune disease, HIV or AIDS, or lymphopenia.

^⁋^ includes therapists, nutritionists, phlebotomists, social workers, and psychologists.

**Table S3**. Characteristics of participating healthcare personnel, by year of enrolment – 2018-19 to 2019-20.

| **Participant characteristic** | **Year 1 Enrollee (N=521)** | | | **Year 2 Enrollee (N=162)** | | | **P-value*** |
| --- | --- | --- | --- | --- | --- | --- | --- |
|  | **N** | **%** | **(95% CI)** | **N** | **%** | **(95% CI)** |  |
| **Study site** |  |  |  |  |  |  | <0.001 |
| Baylor Scott & White Health System | 295 | 56.6 | (52.3, 60.9) | 23 | 14.2 | (8.8, 19.6) |  |
| Kaiser Permanente Northwest | 226 | 43.4 | (39.1, 47.9) | 139 | 85.8 | (80.4, 91.2) |  |
| **Age in years, mean (SD)** | --- | 46.8 | (9.7) | --- | 47.1 | (9.6) | 0.75 |
| **Age group** |  |  |  |  |  |  | 0.52 |
| 18-44 years | 196 | 37.6 | (33.4, 41.8) | 66 | 40.7 | (33.1, 48.3) |  |
| 45-64 years | 325 | 62.4 | (58.2, 66.5) | 96 | 59.3 | (51.7, 66.8) |  |
| **Sex^†^** |  |  |  |  |  |  | 0.21 |
| Male | 86 | 16.5 | (13.3, 19.7) | 36 | 22.2 | (15.8, 28.6) |  |
| Female | 434 | 83.5 | (80.3, 86.7) | 126 | 77.8 | (71.3, 84.2) |  |
| **Race/ethnicity^†^** |  |  |  |  |  |  | <0.01 |
| White, non-Hispanic | 381 | 73.7 | (69.9, 77.5) | 137 | 84.6 | (79.0, 90.1) |  |
| Black, non-Hispanic | 30 | 5.8 | (3.8, 7.8) | 2 | 1.2 | (0.0, 2.9) |  |
| Hispanic | 69 | 13.3 | (10.4, 16.3) | 10 | 6.2 | (2.5, 9.9) |  |
| Other race, non-Hispanic | 37 | 7.1 | (4.9, 9.4) | 13 | 8.0 | (3.8, 12.2) |  |
| **Educational attainment** |  |  |  |  |  |  | 0.01 |
| High school or less | 37 | 7.1 | (4.9, 9.3) | 8 | 4.9 | (1.6, 8.2) |  |
| Some college / Associate's degree | 220 | 42.2 | (38.0, 46.5) | 52 | 32.1 | (24.9, 39.3) |  |
| Bachelor's degree | 131 | 25.1 | (21.4. 28.9) | 40 | 24.7 | (18.0, 31.3) |  |
| Graduate degree | 133 | 25.5 | (21.8, 29.3) | 62 | 38.3 | (30.8, 45.8) |  |
| **Average number of household contacts, mean (SD)** | --- | 2.1 | (1.4) | --- | 2.0 | (1.3) | 0.32 |
| **Health characteristics** |  |  |  |  |  |  |  |
| BMI, mean (SD) | --- | 29.4 | (7.0) | --- | 28.2 | (7.2) | 0.06 |
| Cigarette smoker | 24 | 4.6 | (2.8, 6.4) | 8 | 5.0 | (1.6, 8.4) | 0.83 |
| Diagnosed or treated for chronic medical condition | 112 | 21.5 | (18.0, 25.0) | 40 | 25.0 | (18.3, 31.7) | 0.39 |
| Has immunosuppressive condition^§^ | 12 | 2.3 | (1.0, 3.6) | 5 | 3.1 | (0.4, 5.7) | 0.57 |
| Self-rated general health status | 514 | 98.7 | (97.7, 99.6) | 155 | 96.9 | (94.2, 99.6) | 0.17 |
| **Employment characteristics** |  |  |  |  |  |  |  |
| Occupational category**^†^** |  |  |  |  |  |  | <0.001 |
| *Clinical professional* | 372 | 71.4 | (67.5, 75.3) | 48 | 30.2 | (23.0, 37.3) |  |
| Physician | 61 | 11.7 | (8.9, 14.5) | 11 | 6.9 | (3.1, 6.9) |  |
| Dentist | 2 | 0.4 | (0.0, 0.9) | 0 | 0.0 | --- |  |
| Nurse practitioner | 9 | 1.7 | (0.6, 2.8) | 2 | 1.3 | (0.0, 3.0) |  |
| Physician assistant | 4 | 0.8 | (0.0, 1.5) | 0 | 0.0 | --- |  |
| Nurse or midwife | 215 | 41.3 | (37.0, 45.5) | 23 | 14.5 | (9.0, 19.9) |  |
| Allied health professional^⁋^ | 71 | 13.6 | (10.7, 16.6) | 11 | 6.9 | (3.0, 10.9) |  |
| Pharmacist | 10 | 1.9 | (0.7, 3.1) | 1 | 0.6 | (0.0, 1.9) |  |
| *Clinical paraprofessional* | 86 | 16.5 | (13.3, 19.7) | 24 | 15.1 | (9.5, 20.7) |  |
| Technicians | 60 | 11.5 | (8.8, 14.3) | 19 | 11.9 | (6.9. 17.0) |  |
| Medical assistant | 24 | 4.6 | (2.8, 6.4) | 5 | 3.1 | (0.4, 5.9) |  |
| Patient Transporter | 2 | 0.4 | (0.0, 0.9) | 0 | 0.0 | --- |  |
| *Non-clinical support staff* | 28 | 5.4 | (3.4, 7.3) | 82 | 51.6 | (43.8, 59.3) |  |
| Front Desk & Administrative Staff | 26 | 5.0 | (3.1, 6.9) | 42 | 26.4 | (19.5, 33.3) |  |
| Research personnel | 2 | 0.4 | (0.0, 0.9) | 40 | 25.1 | (18.4, 31.9) |  |
| *Other* | 35 | 6.7 | (4.6, 8.9) | 5 | 3.1 | (0.4, 5.9) |  |
| **Frequency of vaccination**** |  |  |  |  |  |  | 0.01 |
| Consistent vaccinator | 438 | 84.1 | (80.9, 87.2) | 120 | 74.1 | (67.3, 80.8) |  |
| Inconsistent vaccinator | 83 | 15.9 | (12.8, 19.1) | 42 | 25.9 | (19.2, 32.7) |  |
| Vaccinated 4/5 seasons | 35 | 6.7 | (4.5, 8.9) | 31 | 19.1 | (13.1, 25.2) |  |
| Vaccinated <4/5 seasons | 48 | 9.2 | (6.7, 11.7) | 11 | 6.8 | (2.9, 10.7) |  |

* P-value derived from Chi-squared test for categorical variables and Kruskal-Wallis test for continuous variables

† 1 individual was missing information on sex, 4 individuals were missing information on race/ethnicity, and 3 were missing occupation information.

§ immunosuppressive conditions included a diagnosis of cancer with chemotherapy or radiation treatment, organ transplant, diagnosis of autoimmune disease, HIV or AIDS, or lymphopenia.

⁋ includes therapists, nutritionists, phlebotomists, social workers, and psychologists

** A “consistent” vaccinator was defined as a participant who received an influenza vaccine during all five influenza seasons preceding interview based on information in their HR, EMR, or vaccine register; an "inconsistent" vaccinator was unvaccinated for at least one season during the five seasons preceding interview.

**Table S4.** Characteristics of participating healthcare personnel, by study site – 2018-19 to 2019-20.

| **Characteristic** | **Baylor Scott and White Health System (N=318)** | | | **Kaiser Permanente Northwest (N=365)** | | | **P-value*** |
| --- | --- | --- | --- | --- | --- | --- | --- |
|  | **N** | **%** | **(95% CI)** | **N** | **%** | **(95% CI)** |  |
| **Age in years, mean (SD)** | --- | 45.9 | (10.4) | --- | 47.7 | (8.9) | 0.05 |
| **Age group** |  |  |  |  |  |  | 0.53 |
| 18-44 years | 126 | 39.6 | (34.2, 45.0) | 136 | 37.3 | (32.3, 42.2) |  |
| 45-64 years | 192 | 60.4 | (55.0, 65.8) | 229 | 62.7 | (57.8, 67.7) |  |
| **Sex^†^** |  |  |  |  |  |  | 0.07 |
| Male | 48 | 15.1 | (11.1, 19.0) | 74 | 20.3 | (16.2, 24.5) |  |
| Female | 270 | 84.9 | (81.0, 88.9) | 290 | 79.7 | (75.5, 83.8) |  |
| **Race/ethnicity^†^** |  |  |  |  |  |  | <0.001 |
| White, non-Hispanic | 214 | 67.5 | (62.3, 72.7) | 304 | 84.0 | (80.2, 87.8) |  |
| Black, non-Hispanic | 28 | 8.8 | (5.7, 11.9) | 4 | 1.1 | (0.0, 2.2) |  |
| Hispanic | 65 | 20.5 | (16.0, 25.0) | 14 | 3.9 | (1.9, 5.8) |  |
| Other race, non-Hispanic | 10 | 3.1 | (1.2, 5.1) | 40 | 11.0 | (7.8, 14.3) |  |
| **Educational attainment** |  |  |  |  |  |  | <0.001 |
| High school or less | 37 | 11.6 | (8.1, 15.2) | 8 | 2.2 | (0.7, 3.7) |  |
| Some college / Associate's degree | 179 | 56.3 | (50.8, 61.7) | 93 | 25.5 | (21.0, 30.0) |  |
| Bachelor's degree | 66 | 20.7 | (16.3, 25.2) | 105 | 28.8 | (24.1, 33.4) |  |
| Graduate degree | 36 | 11.3 | (7.8, 14.8) | 159 | 43.6 | (38.5, 48.7) |  |
| **Health characteristics** |  |  |  |  |  |  |  |
| BMI, mean (SD) | --- | 30.5 | (6.9) | --- | 27.9 | (6.9) | <0.001 |
| Cigarette smoker**^†^** | 17 | 5.3 | (2.9, 7.8) | 15 | 4.1 | (2.1, 6.2) | 0.45 |
| Diagnosed or treated for chronic medical condition**^†^** | 56 | 17.6 | (13.4, 21.8) | 96 | 26.4 | (21.9, 31.0) | 0.01 |
| Has immunosuppressive condition^§^ | 3 | 0.9 | (0.0, 2.0) | 14 | 3.8 | (1.9, 5.8) | 0.01 |
| Self-rated general health status good or better**^†^** | 313 | 98.4 | (97.1, 99.8) | 356 | 98.1 | (96.6, 99.5) | 0.72 |
| **Employment characteristics** |  |  |  |  |  |  |  |
| Occupational category**^†^** |  |  |  |  |  |  | <0.001 |
| *Clinical professional* | 192 | 60.4 | (55.0, 65.8) | 228 | 62.9 | (58.0, 68.0) |  |
| Physician | 12 | 3.8 | (1.7, 5.9) | 60 | 16.6 | (12.7, 20.4) |  |
| Dentist | 0 | 0 | --- | 2 | 0.5 | (0.0, 1.3) |  |
| Nurse practitioner | 4 | 1.3 | (0.0, 2.5) | 7 | 1.9 | (0.5, 3.3) |  |
| Physician assistant | 2 | 0.6 | (0.0, 1.5) | 2 | 0.5 | (0.0, 1.3) |  |
| Nurse or midwife | 138 | 43.4 | (37.9, 48.9) | 100 | 27.6 | (23.0, 32.2) |  |
| Allied health professional^⁋^ | 34 | 10.7 | (7.3, 14.1) | 48 | 13.3 | (9.7, 16.8) |  |
| Pharmacist | 2 | 0.6 | (0.0, 1.5) | 9 | 2.5 | (0.9, 4.1) |  |
| *Clinical paraprofessional* | 74 | 23.3 | (18.6, 27.9) | 36 | 9.9 | (6.8, 13.0) |  |
| Technicians | 55 | 17.3 | (13.1, 21.5) | 24 | 6.6 | (4.1, 9.2) |  |
| Medical assistant | 17 | 5.3 | (2.9, 7.8) | 12 | 3.3 | (1.5, 5.2) |  |
| Patient Transporter | 2 | 0.6 | (0.0, 1.5) | 0 | 0.0 | --- |  |
| *Non-clinical support staff* | 31 | 9.7 | (6.5, 13.0) | 79 | 21.8 | (17.5, 26.1) |  |
| Front Desk & Administrative Staff | 30 | 9.4 | (6.2, 12.6) | 38 | 10.5 | (7.3, 13.7) |  |
| Research personnel | 1 | 0.3 | (0.0, 0.9) | 41 | 11.3 | (8.1, 14.6) |  |
| *Other* | 21 | 6.6 | (3.9, 9.3) | 19 | 5.2 | (2.9, 7.5) |  |
| **Frequency of vaccination**** |  |  |  |  |  |  | 0.72 |
| Consistent vaccinator | 258 | 81.1 | (76.8, 85.4) | 300 | 82.2 | (78.3, 86.1) |  |
| Inconsistent vaccinator | 60 | 18.9 | (14.5, 23.2) | 65 | 17.8 | (13.9, 21.7) |  |
| 4/5 seasons | 25 | 7.9 | (4.9, 10.8) | 41 | 11.2 | (8.0, 14.5) |  |
| <4/5 seasons | 35 | 11.0 | (7.5, 14.5) | 24 | 6.6 | (4.0, 9.1) |  |

* P-value derived from Chi-squared test for categorical variables and Kruskal Wallis test for continuous variables

†1 participant was missing information on sex, 4 were missing race/ethnicity, 2 were missing smoking status, 2 were missing physical activity, 2 were missing chronic disease diagnosis, 2 were missing self-reported general health, and 3 were missing information on occupation.

§ immunosuppressive conditions included a diagnosis of cancer with chemotherapy or radiation treatment, organ transplant, diagnosis of autoimmune disease, HIV or AIDS, or lymphopenia.

^⁋^ includes therapists, nutritionists, phlebotomists, social workers, and psychologists

** A consistent vaccinator was defined as a participant who received an influenza vaccine all five seasons preceding the interview based on information in their HR, EMR, or vaccine Immunization Information System; an "inconsistent" vaccinator missed an influenza vaccination for at least one season during the five seasons preceding interview**.**

**Table S5.** Characteristics of participating healthcare personnel, by occupational category – 2018-19 to 2019-20.

| **Characteristic** | **Clinical professional (N=420)** | | | **Clinical Paraprofessional (N=110)** | | | **Non-Clinical Support Staff (N=110)** | | | **P-value*** |
| --- | --- | --- | --- | --- | --- | --- | --- | --- | --- | --- |
|  | N | % | (95% CI) | N | % | (95% CI) | N | % | (95% CI) |  |
| **Study site** |  |  |  |  |  |  |  |  |  | <0.001 |
| Baylor Scott & White | 192 | 45.7 | (40.9, 50.5) | 74 | 67.3 | (58.5, 76.1) | 31 | 28.2 | (19.7, 36.6) |  |
| Kaiser Permanente Northwest | 228 | 54.3 | (49.5, 59.1) | 36 | 32.7 | (23.9, 41.5) | 79 | 71.8 | (63.4, 80.2) |  |
| **Age in years, mean (SD)** | --- | 46.5 | (9.5) | --- | 45.8 | (10.9) | --- | 48.3 | (9.2) | 0.16 |
| **Age group** |  |  |  |  |  |  |  |  |  | 0.10 |
| 18-44 years | 168 | 40.0 | (35.3, 44.7) | 49 | 44.5 | (35.2, 53.9) | 34 | 30.9 | (22.3, 39.6) |  |
| 45-64 years | 252 | 60.0 | (55.3, 64.7) | 61 | 55.5 | (46.1, 64.8) | 76 | 69.1 | (60.4, 77.7) |  |
| **Sex^†^** |  |  |  |  |  |  |  |  |  | 0.15 |
| Male | 67 | 16.0 | (12.5, 19.5) | 22 | 20 | (12.5, 27.5) | 26 | 23.6 | (15.7, 31.6) |  |
| Female | 352 | 84.0 | (80.5, 87.5) | 88 | 80 | (72.5, 87.5) | 84 | 76.4 | (68.4, 84.3) |  |
| **Race/ethnicity^†^** |  |  |  |  |  |  |  |  |  | <0.001 |
| White, non-Hispanic | 334 | 80.1 | (76.3, 83.9) | 69 | 62.7 | (53.7, 71.8) | 84 | 76.4 | (68.4, 84.3) |  |
| Black, non-Hispanic | 17 | 4.1 | (2.2, 6.0) | 8 | 7.3 | (2.4, 12.1) | 5 | 4.5 | (0.6, 8.4) |  |
| Hispanic | 32 | 7.7 | (5.1, 10.2) | 28 | 25.5 | (17.3, 33.6) | 14 | 12.7 | (6.5, 19.0) |  |
| Other race, non-Hispanic | 34 | 8.1 | (5.5, 10.8) | 5 | 4.5 | (0.6, 8.4) | 7 | 6.4 | (1.8, 10.9) |  |
| **Educational attainment** |  |  |  |  |  |  |  |  |  | <0.001 |
| High school or less | 9 | 2.1 | (0.7, 3.5) | 18 | 16.4 | (9.4, 23.3) | 6 | 5.5 | (1.2, 9.7) |  |
| Some college / Associate's degree | 139 | 33.1 | (28.6, 37.6) | 75 | 68.2 | (59.5, 76.9) | 38 | 34.5 | (25.6, 43.5) |  |
| Bachelor's degree | 126 | 30.0 | (25.6, 34.4) | 13 | 11.8 | (5.8, 17.9) | 27 | 24.5 | (16.5, 32.6) |  |
| Graduate degree | 146 | 34.8 | (30.2, 23.3) | 4 | 3.6 | (0.1, 7.1) | 39 | 35.5 | (26.5, 44.4) |  |
| **Health characteristics** |  |  |  |  |  |  |  |  |  |  |
| BMI, mean (SD) | --- | 28.3 | (6.5) | --- | 31.1 | (7.6) | --- | 29.6 | (7.6) | <0.001 |
| Cigarette smoker | 17 | 4.0 | (2.1, 5.9) | 3 | 2.7 | (0.0, 5.8) | 9 | 8.3 | (3.1, 13.4) | 0.10 |
| Physical activity in the last 30 days | 363 | 86.4 | (83.1, 89.7) | 89 | 80.9 | (73.5, 88.3) | 87 | 79.8 | (72.3, 87.4) | 0.13 |
| Diagnosed or treated for chronic medical condition in past 12 months | 97 | 23.1 | (19.1, 27.1) | 20 | 18.2 | (10.9, 25.4) | 27 | 24.8 | (16.6, 32.9) | 0.45 |
| Has immunosuppressive condition^§^ | 11 | 2.6 | (1.1, 4.1) | 2 | 1.8 | (0.0, 4.3) | 3 | 2.7 | (0.0, 5.8) | 0.88 |
| Self-rated general health status | 418 | 99.5 | (98.9, 100) | 107 | 97.3 | (94.2, 100) | 104 | 95.4 | (91.5, 99.3) | 0.01 |
| **Employment characteristics** |  |  |  |  |  |  |  |  |  |  |
| Number of hours worked, mean (SD) | --- | 38.1 | (9.5) | --- | 42 | (11.2) | --- | 40.4 | (8.8) | <0.001 |
| Number of hours with patient contact, mean (SD) | --- | 28.0 | (12.9) | --- | 27.5 | (16.8) | --- | 26.9 | (15.5) | <0.001 |
| **Frequency of vaccination^⁋^** |  |  |  |  |  |  |  |  |  | 0.01 |
| Consistent vaccinator | 357 | 85.0 | (81.6, 88.4) | 87 | 79.1 | (71.5, 86.7) | 81 | 73.6 | (65.4, 81.9) |  |
| Inconsistent vaccinator | 63 | 15.0 | (11.6, 18.4) | 23 | 20.9 | (13.3, 28.5) | 29 | 26.4 | (18.1, 34.6) |  |
| 4/5 seasons | 33 | 7.9 | (5.3, 10.4) | 13 | 11.8 | (5.8, 17.9) | 16 | 14.5 | (7.9, 21.1) |  |
| <4/5 seasons | 30 | 7.1 | (4.7, 9.6) | 10 | 9.1 | (3.7, 14.5) | 13 | 11.8 | (5.8, 17.9) |  |

* P-value derived from Chi-squared test for categorical variables and Kruskal-Wallis test for continuous variables

† 1 individual was missing information on sex and 3 individuals were missing information on race/ethnicity.

§ immunosuppressive conditions included a diagnosis of cancer with chemotherapy or radiation treatment, organ transplant, diagnosis of autoimmune disease, HIV or AIDS, or lymphopenia.

^⁋^ A “consistent” vaccinator was defined as a participant who received an influenza vaccine during all five influenza seasons preceding interview based on information in their HR, EMR, or vaccine register; an "inconsistent" vaccinator was unvaccinated for at least one season during the five seasons preceding interview.

**Table S6**. Characteristics of healthcare personnel (N=683) with known and uncertain self-reported influenza vaccination status for the five influenza seasons preceding interview – 2018-19 to 2019-20.

| **Characteristic** | **Participants with Known Self-Reported Vaccination Status (N=561)** | | | **Participants with Uncertain Self-Reported Vaccination Status (N=122)** | | | **P-value^†^** |
| --- | --- | --- | --- | --- | --- | --- | --- |
|  | **n** | **%** | **(95% CI)** | **n** | **%** | **(95% CI)** |  |
| **Study site** |  |  |  |  |  |  | 0.66 |
| Baylor Scott & White Health System | 259 | 46.2 | (42.0, 50.3) | 59 | 48.4 | (39.5, 57.3) |  |
| Kaiser Permanente Northwest | 302 | 53.8 | (49.7, 58.0) | 63 | 51.6 | (42.7, 60.5) |  |
| **Age in years, mean (SD)** | --- | 47.1 | (9.6) | --- | 45.7 | (10.0) | 0.21 |
| **Age group** |  |  |  |  |  |  | 0.51 |
| 18-44 years | 212 | 37.8 | (33.8, 41.8) | 50 | 41 | (32.2, 49.7) |  |
| 45-64 years | 349 | 62.2 | (58.2, 66.2) | 72 | 59 | (50.3, 67.8) |  |
| **Sex^§^** |  |  |  |  |  |  | 0.57 |
| Male | 98 | 17.5 | (14.3, 20.7) | 24 | 19.7 | (12.6, 26.7) |  |
| Female | 462 | 82.5 | (79.3, 85.7) | 98 | 80.3 | (73.3, 87.4) |  |
| **Race/ethnicity^§^** |  |  |  |  |  |  | 0.02 |
| White, non-Hispanic | 438 | 78.5 | (75.1, 81.9) | 80 | 66.1 | (57.7, 74.6) |  |
| Black, non-Hispanic | 22 | 3.9 | (2.3, 5.6) | 10 | 8.3 | (3.3, 13.2) |  |
| Hispanic | 58 | 10.4 | (7.9, 12.9) | 21 | 17.3 | (10.6, 24.1) |  |
| Other race, non-Hispanic | 40 | 7.2 | (5.0, 9.3) | 10 | 8.3 | (3.3, 13.2) |  |
| **Educational attainment** |  |  |  |  |  |  | <0.001 |
| High school or less | 23 | 4.1 | (2.5, 5.7) | 22 | 18 | (11.2, 24.9) |  |
| Some college / Associate's degree | 227 | 40.5 | (36.5, 44.5) | 45 | 36.9 | (28.3, 45.5) |  |
| Bachelor's degree | 144 | 25.7 | (22.0, 29.3) | 27 | 22.1 | (14.7, 29.5) |  |
| Graduate degree | 167 | 29.8 | (26.0, 33.6) | 28 | 22.9 | (15.5, 30.4) |  |
| **Health characteristics** |  |  |  |  |  |  |  |
| BMI, mean (SD) | --- | 28.9 | (1.3) | --- | 30.3 | (7.9) | 0.10 |
| Cigarette smoker | 22 | 3.9 | (2.3, 5.5) | 10 | 8.3 | (3.4, 13.3) | 0.04 |
| Diagnosed or treated for chronic medical condition | 131 | 23.3 | (19.8, 26.9) | 21 | 17.5 | (10.7, 24.3) | 0.16 |
| Has immunosuppressive condition^⁋^ | 14 | 2.5 | (1.2, 3.8) | 3 | 2.5 | (0.0, 5.2) | 0.98 |
| Self-rated general health status good or better | 554 | 98.7 | (97.8, 99.7) | 115 | 95.8 | (92.2, 99.4) | 0.03 |
| **Employment characteristics** |  |  |  |  |  |  |  |
| Occupational category**^§^** |  |  |  |  |  |  | <0.001 |
| *Clinical professional* | 365 | 65.3 | (61.3, 69.3) | 55 | 45.5 | (36.6, 54.3) |  |
| Physician | 68 | 12.2 | (9.4, 14.9) | 4 | 3.3 | (0.1, 6.5) |  |
| Dentist | 1 | 0.2 | (0.0, 0.5) | 1 | 0.8 | (0.0, 2.4) |  |
| Nurse practitioner | 11 | 2.0 | (0.8, 3.1) | 0 | 0.0 | --- |  |
| Physician assistant | 4 | 0.7 | (0.0, 1.4) | 0 | 0.0 | --- |  |
| Nurse or midwife | 207 | 37.0 | (33.0, 41.0) | 31 | 25.6 | (17.8, 33.4) |  |
| Allied health professional** | 66 | 11.8 | (9.1, 14.5) | 16 | 13.2 | (7.2, 19.3) |  |
| Pharmacist | 8 | 1.4 | (0.4, 2.4) | 3 | 2.5 | (0.0, 5.3) |  |
| *Clinical paraprofessional* | 80 | 14.3 | (11.4, 17.2) | 30 | 24.8 | (17.1, 32.5) |  |
| Technicians | 60 | 10.7 | (8.2, 13.3) | 19 | 15.7 | (9.2, 22.2) |  |
| Medical assistant | 20 | 3.6 | (2.0, 5.1) | 9 | 7.4 | (2.7, 12.1) |  |
| Patient Transporter | 0 | 0.0 | --- | 2 | 1.7 | (0.0, 3.9) |  |
| *Non-clinical support staff* | 87 | 15.6 | (12.5, 18.6) | 23 | 19.0 | (12.0, 26.0) |  |
| Front Desk & Administrative Staff | 59 | 10.5 | (8.0, 13.1) | 9 | 7.4 | (2.7, 12.1) |  |
| Research personnel | 28 | 5.0 | (3.2, 6.8) | 14 | 11.6 | (5.9, 17.3) |  |
| *Other* | 27 | 4.8 | (3.0, 6.6) | 13 | 10.7 | (5.2, 16.3) |  |
| **Frequency of vaccination**^††^ |  |  |  |  |  |  | <0.001 |
| Consistent vaccinator | 471 | 83.9 | (80.9, 87.0) | 87 | 71.3 | (63.3, 79.3) |  |
| Inconsistent vaccinator | 90 | 16.0 | (13.0, 19.1) | 35 | 28.7 | (20.6, 36.7) |  |
| Vaccinated 4/5 seasons | 49 | 8.7 | (6.4, 11.1) | 17 | 13.9 | (7.8, 20.1) |  |
| Vaccinated <4/5 seasons | 41 | 7.3 | (5.1, 9.5) | 18 | 14.7 | (8.4, 21.1) |  |

*Participants with uncertain self-reported vaccination status either reported not knowing their vaccination status (n=111) or did not indicate their vaccination status on the survey (n=11).

†P-value derived from Chi-squared test for categorical variables and Kruskal Wallis test for continuous variables.

^§^ 1 participant was missing information on sex, 4 were missing information on race/ethnicity, and 3 were missing occupation information.

^⁋^ immunosuppressive conditions included a diagnosis of cancer with chemotherapy or radiation treatment, organ transplant, diagnosis of autoimmune disease, HIV or AIDS, or lymphopenia.

** includes therapists, nutritionists, phlebotomists, social workers, and psychologists.

^††^A “consistent” vaccinator was defined as a participant who received an influenza vaccine during all five influenza seasons preceding interview based on information in their HR, EMR, or vaccine register; an "inconsistent" vaccinator was unvaccinated for at least one season during the five seasons preceding interview.

**Table S7**. Measures of agreement between self-reported vaccination status for healthcare personnel (N=494)* as measured across two consecutive years of interview – 2018-19 to 2019-20.

| **Influenza Season** | **Percentage of Participants with Known Self-Reported Vaccination Status** | | | | **Absolute Difference†** | **Self-Reported Vaccination Status** | | **Absolute Difference^§^** | **Prevalence-adjusted Kappa Coefficient^⁋^** |
| --- | --- | --- | --- | --- | --- | --- | --- | --- | --- |
|  | **As reported in Year 1** | | **As reported in Year 2** | |  | **As reported in Year 1** | **As reported in Year 2** |  |  |
|  | N | % (95% CI) | N | % (95% CI) | % | % (95% CI) | % (95% CI) | % | Coeff. (95% CI) |
| 2017-18 season | 446 | 90.3 | 402 | 81.4 | 8.9 | 97.6 | 99.5 | -1.9 | 94.1 (90.7, 97.5) |
| 2016-17 season | 441 | 89.3 | 403 | 81.6 | 7.7 | 99.2 | 99.7 | -0.5 | 98.9 (97.4, 100) |
| 2015-16 season | 427 | 86.4 | 398 | 80.6 | 5.8 | 98.6 | 98.6 | 0.0 | 98.9 (97.4, 100) |
| 2014-15 season | 417 | 84.4 | 384 | 77.7 | 6.7 | 96.8 | 96.3 | 0.5 | 96.5 (93.8, 99.3) |
| 2013-14 season | 395 | 80.0 | 379 | 76.7 | 3.3 | 95.7 | 94.2 | 1.5 | 92.1 (87.8, 96.3) |

* Includes only year 1 participants who also participated in year 2 data collection

† The absolute difference in the percent of healthcare personnel who were able to recall their vaccination status in the second year of interview vs. the first year of interview

^§^ The absolute difference in the percent of vaccinated healthcare personnel as measured by self-reported vaccination status in the second year of interview vs. the first year of interview

^⁋^ Kappa coefficient describing the degree of agreement between self-reported vaccination status in the first year of interview and the second year of interview.

**Table S8**. Sensitivity analysis of agreement between self-reported and documented influenza vaccination status for healthcare personnel participating in Year 1 only (N=521) – 2018-19.

| **Influenza Season** | **Able to self-report vaccination status** | | **Self-reported vaccination status** | **Documented vaccination status** | **Absolute Difference*** | **Prevalence-adjusted Kappa Coefficient^†^** | **Sensitivity** | **Specificity** | **Positive Predictive Value** | **Negative Predictive Value** |
| --- | --- | --- | --- | --- | --- | --- | --- | --- | --- | --- |
|  | N | % (95% CI) | % (95% CI) | % (95% CI) | % | Coeff. (95% CI) | Value (95% CI) | Value (95% CI) | Value (95% CI) | Value (95% CI) |
| Preceding season | 472 | 90.6 | 97.3 | 97.3 | 0.0 | 89.8 (85.9, 93.8) | 97.4 (95.5, 98.6) | 7.7 (0.2, 36.0) | 97.4 (96.9, 97.8) | 7.7 (1.2, 37.3) |
| 2 seasons prior | 471 | 90.4 | 99.8 | 94.1 | 5.7 | 88.5 (84.3, 92.7) | 100 (99.2, 100) | 3.6 (0.1, 18.3) | 94.3 (93.9, 94.6) | ---^§^ |
| 3 seasons prior | 462 | 88.7 | 99.3 | 95.0 | 4.3 | 90.5 (86.6, 94.4) | 99.8 (98.7, 100) | 8.7 (1.1, 28.0) | 95.4 (94.8, 95.9) | 66.7 (15.8, 95.5) |
| 4 seasons prior | 453 | 86.9 | 98.9 | 90.9 | 8.0 | 84.1 (79.1, 89.1) | 100 (99.1, 100) | 12.2 (4.1, 26.2) | 92.0 (91.1, 92.8) | ---^§^ |
| 5 seasons prior | 438 | 84.1 | 98.4 | 91.1 | 7.3 | 83.6 (78.4, 88.7) | 99.5 (98.2, 99.9) | 12.8 (4.3, 27.4) | 92.1 (91.2, 92.9) | 71.4 (33.4, 92.6) |

*Absolute difference in self-reported vaccination status vs. documented vaccination status

**^†^** Kappa coefficient showing degree of agreement between self-reported and documented influenza vaccination status.

^§^ Unable to estimate based on small numbers (n<2)

**Table S9**. Sensitivity analysis of agreement between self-reported and documented influenza vaccination status during the three years prior to interview among participating healthcare personnel (N=801) – 2018-19 to 2019-20.

| **Influenza Season** | **Able to self-report vaccination status** | | **Self-reported vaccination status** | **Documented vaccination status** | **Absolute Difference*** | **Prevalence-adjusted Kappa Coefficient^†^** | **Sensitivity** | **Specificity** | **Positive Predictive Value** | **Negative Predictive Value** |
| --- | --- | --- | --- | --- | --- | --- | --- | --- | --- | --- |
|  | N | % (95% CI) | % (95% CI) | % (95% CI) | % | Coeff. (95% CI) | Value (95% CI) | Value (95% CI) | Value (95% CI) | Value (95% CI) |
| Preceding season | 717 | 89.5 | 97.1 | 97.5 | -0.4 | 90.2 (87.1, 93.4) | 99.7 (98.9, 100) | 54.3 (36.7, 71.2) | 97.7 (96.7, 98.4) | 90.5 (69.7, 97.5) |
| 2 seasons prior | 709 | 88.5 | 99.4 | 92.5 | 6.9 | 93.8 (91.2, 96.3) | 99.4 (98.5, 99.8) | ---^§^ | 97.5 (97.4, 97.5) | ---^§^ |
| 3 seasons prior | 696 | 86.9 | 97.8 | 94.1 | 3.7 | 90.8 (87.7, 93.9) | 97.8 (96.4, 98.8) | ---^§^ | 97.5 (97.5, 97.5) | ---^§^ |

*Absolute difference in self-reported vaccination status vs. documented vaccination status

**^†^** Kappa coefficient showing degree of agreement between self-reported and documented influenza vaccination status.

^§^ Unable to estimate due to small numbers (n<2).

**Table S10.** Sensitivity analysis of agreement between self-reported and documented influenza vaccination status for healthcare personnel who did not receive an influenza vaccine in all five seasons preceding enrolment (i.e., “inconsistent vaccinators*) (N=125) – 2018-19 to 2019-20.

| **Influenza Season** | **Able to self-report influenza vaccine status** | | **Self-reported vaccination status** | **Documented vaccination status** | **Absolute % Difference**† | **Prevalence-adjusted Kappa Coefficient**^§^ | **Sensitivity** | **Specificity** | **Positive Predictive Value** | **Negative Predictive Value** |
| --- | --- | --- | --- | --- | --- | --- | --- | --- | --- | --- |
|  | N | % (95% CI) | % (95% CI) | % (95% CI) | % | Coeff. (95% CI) | Value (95% CI) | Value (95% CI) | Value (95% CI) | Value (95% CI) |
| Preceding season | 106 | 84.8 | 98.1 | 87.7 | 10.4 | 75.5 (63.0, 88.0) | 98.9 (94.1, 100) | 7.7 (0.2, 36.0) | 88.5 (86.7, 90.0) | 50.0 (6.2, 93.8) |
| 2 seasons prior | 105 | 84.0 | 97.1 | 58.1 | 39.0 | 21.9 (3.2, 40.6) | 100 (94.1, 100) | 6.8 (1.4, 18.7) | 59.8 (57.9, 61.7) | ---^⁋^ |
| 3 seasons prior | 100 | 80.0 | 92.0 | 72.0 | 20.0 | 48.0 (30.8, 65.2) | 95.8 (88.3, 99.1) | 17.9 (6.1, 36.9) | 75.0 (71.5, 78.2) | 62.5 (29.9, 86.7) |
| 4 seasons prior | 95 | 76.0 | 89.5 | 53.7 | 35.8 | 15.8 (-4.1, 35.7) | 94.1 (83.8, 98.9) | 15.9 (6.6, 30.1) | 56.5 (52.9, 60.0) | 70.0 (39.1, 89.5) |
| 5 seasons prior | 93 | 74.4 | 83.9 | 36.6 | 47.3 | -3.2 (-23.5, 17.1) | 94.1 (80.3, 99.3) | 22.0 (12.3, 34.7) | 41.0 (37.2, 44.9) | 86.7 (60.9, 96.4) |

*An "inconsistent" vaccinator was unvaccinated for at least one season during the five seasons preceding interview.

†The absolute difference in the percent of vaccinated healthcare personnel as measured by self-reported vs. documented vaccination status.

^§^ Kappa coefficient describing the degree of agreement between self-reported vaccination status in the first year of interview and the second year of interview.

^⁋^ Unable to estimate based on small numbers (n<2)

**Figure S1.** Percent of healthcare personnel self-reporting their influenza vaccination status for the five preceding influenza seasons, by frequency of vaccination – 2018-19 to 2019-20.

*A “consistent” vaccinator was defined as a participant who received an influenza vaccine during all five influenza seasons preceding interview based on information in their HR, EMR, or vaccine register; an "inconsistent" vaccinator was unvaccinated for at least one season during the five seasons preceding interview.
